# Parents’ experiences of information-seeking and decision-making regarding complementary medicine for children with autism spectrum disorder: a qualitative study

**DOI:** 10.1186/s12906-019-2805-0

**Published:** 2020-01-13

**Authors:** Caroline A. Smith, Chloe Parton, Marlee King, Gisselle Gallego

**Affiliations:** 1 NICM Health Research Institute, Western Sydney University, Campbelltown Campus, Locked Bag 1797, Penrith, Sydney, NSW 2571 Australia; 2 Translational Health Research Institute, Western Sydney University, Sydney, NSW Australia; 3 School of Medicine, Western Sydney University, Sydney, NSW Australia; 4 School of Medicine, University of Notre Dame, Sydney, NSW Australia

**Keywords:** Parental decision-making, Complementary medicine, Autism spectrum disorder

## Abstract

**Background:**

Complementary and alternative medicine and therapies (CAM) are widely used by parents of children with autism spectrum disorder (ASD). However, there is a gap in our understanding of how and why parents of children with ASD make decisions about CAM treatment, and how “evidence” influences their decision-making. The aim of this study was to explore views and perspectives on CAM decision-making among parents of children with ASD in Australia.

**Methods:**

Semi-structured interviews were conducted with parents of children with ASD (18 years and under) who were living in Australia. The interviews were digitally recorded, transcribed and then analysed using thematic analysis.

**Results:**

Twenty-one parents were interviewed (20 women and one man). The mean age of participants was 43 years, (SD = 5.12 years), the majority of whom were born in Australia (71%), and almost half (43%) had a bachelor degree or higher. Three main themes were identifiedin the thematic analysis. First theme was ‘Parents’ experiences of researching CAM treatments, the second theme was, “Navigating CAM information and practices”, which comprises of the subthemes: Assessing information on CAM treatments’ What counts as ‘evidence’? and Assessing the impact of CAM treatments on the child - What counts as effective?, and the final theme was, “Creating a central and trustworthy source about CAM”.

Across themes parents’ CAM decision-making was described as pragmatic, influenced by time, cost, and feasibility. Parents also reported that information on CAM was complex and often conflicting, and the creation of a centralised and reliable source of information on CAM was identified as a potential solution to these challenges.

**Conclusion:**

The development of evidence-based information resources for parents and supporting CAM health literacy may assist with navigating CAM decision-making for children’s with ASD.

## Background

Autism spectrum disorder (ASD) is characterised by impairments in communication skills, social interactions and repetitive, restricted, and stereotyped patterns of interests and behaviours [1]. According to the 2015 Survey of Disability, Ageing and Carers, around 164,000 Australians have a diagnosis of autism [2]. In addition, the prevalence of parent-reported ASD in the Longitudinal Study of Australian Children was reported as 2.5% (95% confidence interval = [2.0, 3.0]) for children at birth compared to 1.5% (95% confidence interval = [1.2, 2.0]) for children in kindergarten [3]. Treatment approaches for ASD focus on behavioural interventions, speech therapy, occupational therapy and educational interventions. There has been increasing evidence over the past decade to suggest that complementary and alternative medicine (CAM) is widely used by parents with children who have ASD [4–8]. However, little is known about how parents perceive and negotiate information on CAM as part of their decision making regarding its use with children.

The United States Institute of Medicine defined CAM as “healing resources that encompasses all health systems, modalities, and practices and their accompanying theories and beliefs, other than those intrinsic to the politically dominant health system of a particular society or culture in a given historical period” [9]. CAM includes all such practices and ideas self-defined by their users as preventing or treating illness or promoting health and well-being. International studies have reported a lifetime prevalence of CAM use, ranging from 39 to 92% [10–12]. The variation in prevalence is due to the differing definitions of CAM used in studies. The high use of CAM among children with ASD and the mixed evidence regarding the efficacy of these treatments [8] suggests there is a need to understand how parents make decisions regarding their child’s CAM use.

Commonly used CAM among children with ASD reported in the literature [7, 13] include: special diets (e.g. Gluten-Free, Casein-Free Diet (GFCF)), nutritional supplements, music therapy, magnetic therapy, acupuncture, craniosacral therapy, chelation, secretin, hyperbaric oxygen therapy, chiropractic care, melatonin, massage and qi gong (a form of traditional Chinese exercise). A survey conducted in Australia with parents of children with ASD found that the most common type of CAM used was fish oil [7]. Reviews however have found that the methodological quality of studies are low and that conclusions cannot be drawn on the effectiveness of many CAM modalities [13, 14]. There is evidence to suggest that melatonin is effective for the treatment for sleeping disturbances associated with ASD [13]. In addition, acupuncture [15–18] carnosine and qi gong [19] may also improve developmental and behavioural aspects of ASD. However, there is insufficient evidence regarding modified diets, hyperbaric oxygen therapy, immune therapy, and vitamin and fatty acid supplementation [13]. Overall, evidence of the effectiveness of these modalities is inconsistent and further randomised controlled trials are required to evaluate the effectiveness and safety of CAM for treating ASD with children.

### Previous research

An online survey conducted by Hopf et al. [20] in the United States found that many CAM treatments were perceived as effective by parents for the management of ASD. This is despite research evidence on the effectiveness of CAM for ASD management that is frequently conflicting and inconsistent, or lacking a scientific rationale [8, 21]. Christon et al. [5] conducted an online survey that found parents entered into CAM treatment with high expectations, however they differed considerably in their assessment of whether CAM helped their child. An Australian qualitative study examined parents’ decision-making processes and information preferences for treatments following their child’s diagnosis of ASD (including all treatment options not only CAM) and found that consideration of the effectiveness or evidence supporting interventions was largely absent [22]. To date, much of the research related to the use of CAM by parents with children who have ASD has been conducted in North America using surveys [5, 20, 23]. In Australia, studies have explored information sources, motivations and reasoning among parents for initiating dietary interventions and nutritional supplements for their child with ASD [24]. The main sources of information reported by parents in this qualitative study included: books, the internet, complementary health practitioners, and other parents of children with ASD. In addition, the parents commented on the significant time they spent searching for information on the internet, and the need for more information on CAM. Further, complementary medicine decision-making was often complex given the frequently uncertain and limited evidence available to parents. Many parents who use CAM with their children are likely to lack adequate information to assist them in minimising risk and enable safe and effective CAM use [4, 25]. In addition, parents are often managing a complex array of treatments and therapies for their child’s ASD, including pharmacological, dietary, behavioral, and educational interventions [26]. Furthermore, perceived benefits from CAM treatments for ASD can be based on personal values, beliefs and expectations [26], and individuals are known to make decisions based on limited anecdotal information [4, 25].

Perspectives on what is meant by the term ‘evidence’ differ among researchers and national bodies. A review by Lomas et al. [27], found that most researchers view evidence scientifically and define it by its methodology while decision-makers view evidence informally and define it by its relevance. For example, the Canadian Health Services Research Foundation defines evidence as: *Information that comes closest to the facts of a matter. The form it takes depends on the context. The findings of high-quality, methodologically appropriate research are the most accurate evidence. Because research is often incomplete and sometimes contradictory or unavailable, other kinds of information are necessary supplements to or stand-ins for research. The evidence base for a decision is the multiple forms of evidence combined to balance rigour with expedience – while privileging the former over the latter* (page 9) [28].

Other organizations such as the Australian National Health and Medical Research Council (NHMRC) and the Oxford Centre for Evidence Based Medicine also describe the hierarchy of evidence (Level I Systematic review to level IV Case series). Individuals using CAM may seek information from multiples sources (friends/families, the internet, print material) and may believe that non research based information provides proof of an effect equivalent to scientific evidence. Currently, little is known about how and why parents of children with ASD make decisions about CAM treatment, and how research “evidence” influences their decision-making. With the widespread use of CAM among parents of children with ASD and limited understanding of how parents make decisions to use CAM, greater knowledge of the role of information is important to enable parents to best manage the health of their children. Hence, the main aim of this study was to explore values and philosophical beliefs and perspectives on complementary and alternative medicine (CAM) decision-making among parents of children with autism spectrum disorder (ASD) in Australia. In particular the study asked: a) what are parents’ experiences of researching treatments and assessing CAM-related information, and b) how do parents understand what constitutes “evidence” and “effectiveness” of CAM treatments.

## Methods

### Design

An exploratory qualitative study was conducted. Twenty-one parents of a child with ASD took part in semi-structured interviews. Interview transcripts were analysed using an inductive descriptive thematic analysis [29].

### Participants and recruitment

Convenience sampling [30] was used to was used to identify parents of a child with ASD. The eligibility criteria for the study included parents who have a child with ASD (under 18 years of age), had used or were thinking of using CAM, were able to provide informed consent for participation in the study and able to communicate in English. Several channels were used to recruit participants who were primarily based at Schools and support groups in Western Sydney, Australia. Referrals to the study were also made by occupational and speech therapist practitioners. Firstly, an invitation to participate in the study was distributed via email to non-government organisations (NGOs) that provide services to children with ASD and schools in the area. For institutions indicating a positive response, study details were then listed in the newsletters and websites of these organisations. Secondly, a flyer was displayed in public venues used by parents of children with ASD such as advocacy groups, the University and public libraries. Finally, the flyer was also displayed on social media sites associated with ASD support groups and CAM interest groups based in Australia. Parents who met the eligibility criteria and who were interested in participating in an interview contacted the researchers directly. Thematic saturation was reached when additional interviews ceased to provide new information [31]. As such, recruitment ended after 21 interviews.

### Data collection

Data were collected via individual semi-structured interviews by author CP, between August and November 2014. As part of using a semi-structured approach, the format and wording of the interview questions were used flexibly according to individual participant responses in order to gather in-depth accounts of experience [32]. As parents of children with ASD have many demands on their time, to ensure that participation was accessible both telephone and face-to-face interviews were offered at a time that suited the parent. Seventeen parents elected to be interviewed over the telephone and four parents elected to be interviewed face-to-face at either a school for children with ASD or the University. A semi-structured interview guide was developed based on a review of the literature and discussions within the research team and people working with children with ASD (see Table 1). The participants were asked questions about the types and sources of information used, including what types of information are accepted as evidence regarding CAM. As part of adopting a semi-structured approach, questions were used conversationally and flexibly to suit the context of the individual participant, which assisted in the development of rapport and gathering more in-depth responses [33]. At the end of the interview parents were also asked to complete a brief questionnaire to collect demographic information (age, employment status, income) as well as details about their CAM use. Parents received a gift card to acknowledge their time and contribution to the study. All interviews were digitally recorded and transcribed verbatim. Some researchers have argued that the use of telephone interviews can limit rapport building and the quality of responses through the lack of embodied visual cues compared to face-to-face interviews [34, 35]. However, in this study no differences were observed in the depth of content or quality of responses depending on whether interviews were conducted face-to-face or over the telephone. Furthermore, the author (CP) who carried out these interviews has extensive experience conducting both telephone and face-to-face qualitative interviews, which may have assisted in the building of rapport to enable rich responses regardless of communication mode. Each parent was allocated a pseudonym and all data were de-identified (names, places were removed) prior to analysis.

**Table 1:** Interview guide

1. Tell us about your experience with complementary medicine? Prompts: use in ASD and frequency of use	
2. How did you find out about CAM therapies? Prompt: source of information (from whom and where information was sourced)	
3. Which of these types of information did you like best?	
4. What convinces you to try a CAM therapy?	
5. How did you decide which information was useful?	
6. How would you describe the quality of the information?	
7. How do you know when information is truthful?	
8. How do you deal with conflicting information about CAM?	
9. What were the most important factors that influence your decision to use or not use CAM?	

### Data analysis

An inductive, descriptive thematic analysis was conducted on the interview transcripts, which followed several steps [29]. Firstly, transcripts were read and re-read to establish familiarity with the data. Secondly, initial codes that captured features of interest in the data relevant to the research questions were identified (author CP) [36]. A coding framework was then developed by collating initial codes together to create candidate codes that meaningfully described the overall patterns of participant responses in the data (authors CP & GG). Transcripts were reviewed to make sure that participant responses were captured by the coding framework (authors CP & GG), and then the transcripts were then coded line-by-line to collate all instances of patterns that were identified in the data (authors CP and MK). Finally, candidate codes were then collapsed to produce higher order themes that were then read for patterns of similarity and divergence within and across each theme (CP, GG & CS). Data management was supported using the qualitative software programme NVivo (QSR International Pty Ltd. V.9). The analysis presented in this paper focuses on parents’ experiences of researching treatments and assessing CAM-related information and practices through their understandings of what constitutes “evidence” and “effectiveness”.

Participant’s demographic data were analysed using STATA 11.0 for Windows (StataCorp LP, College Station, TX). Descriptive statistics were used to summarise these data.

### Ethical approval

This study was approved by Human Research Ethics Committee of [removed for peer-review] University (H10769)*.* Written consent was obtained from all study participants.

## Results

Twenty-one parents took part in the study. The average interview duration was 49 min (range = 29–90 min). Of the 21 parents interviewed, 20 were women. The participants had a mean age of 43 years (range: 31–54 years; SD = 5.12 years), the majority were born in Australia (71%) and almost half (43%) had a bachelor degree or higher. The participants’ socio-demographic characteristics are described in full in Table 2.

**Table 2:** Socio-demographic characteristics of participants

Characteristic	Sample % (n)
Gender	
Female	95.2 (20)
Age	
Mean, years	43.1
Range, years	31–54
Age of the parents’ children with ASD	
Mean, years	8.7
Range, years	3 to 17
Age of ASD diagnosis	
Mean, years	4.0
Range, years	2 to 11
Education	
Completed high school	4.8 (1)
Post-school qualification (e.g. certificate or diploma)	38.1 (8)
Bachelor degree or higher	42.9 (9)
Other^a^	14.3 (3)
Employment	
Not in the paid work force	23.8 (5)
Full-time	9.5 (2)
Part-time	38.1 (8)
Other^b^	28.6 (6)
Marital status	
Never married	4.8 (1)
Married / De facto	81 (17)
Divorced	4.8 (1)
Separated, but not divorced	9.5 (2)
Country born	71.4 (15)
Australia	
Private health insurance	
Yes	66.7 (14)
Language spoken at home	
English	90.5 (19)
Annual household income-gross (*N* = 20)	
Less than $20,000 per year	4.8 (1)
$20,000–$39,999 per year	14.3 (3)
$40,000–$59,999 per year	9.52 (2)
$60,000–$75,999 per year	9.52 (2)
$80,000–$99,999 per year	14.3 (3)
$100,000–$149,999 per year	28.6 (6)
$150,000 or more	14.3 (3)

^a^Other: currently studying, ^b^other: studying, employed on a casual basis

The ages of the parents’ children with ASD ranged from 3 to 17 years (Mean = 8.7 years, SD 3.7 years) at the time of the interview. While most parents had one child with ASD (*n* = 17), two parents had two children, and two parents had three children with ASD. The children were identified by their parents as being across the spectrum with descriptions including “high functioning”, “severely autistic” or as “a child with Asperger’s Syndrome”. A range of symptoms experienced by children was reported, including food intolerances, difficulty regulating emotion, difficulty sleeping, sensory sensitivity, difficulties gauging appropriate social behaviour and a lack of verbal communication. The CAM treatment methods that were used by parents included naturopathy, nutritional supplements, diet changes and acupuncture (see Table 3). Finally, parents were also asked in the questionnaire if they had mentioned the use of CAM to a medical practitioner involved in their children’s care, and the majority (86% *n* = 18) reported that they had mentioned it. However, of those, three (14%) only mentioned diet changes but did not report the use of other therapies.

**Table 3:** Complementary medicines and therapies reported by study participants

Modality^a^	*N* = 21	%
Diet		
Diary free	10	47.6
Gluten free	12	57.1
Casein free	2	9.5
Elimination diet	2	9.5
Other lactose free, phenol diet	3	14.2
Nutritional supplements		
Multi-vitamins (B, C, D, E)	18	85.7
Fish oils	10	47.6
Melatonin	6	28.5
Other unspecified	2	9.5
Minerals (zinc, magnesium, selenium)	12	57.1
Mind-body practices		
Mindfulness	1	4.7
Massage	5	23.8
Bowen Therapy	3	14.2
Kinesiology	4	19.0
Chiropractic	5	25.0
Cranial sacral	1	5.0
Osteopath	2	9.5
Aromatherapy	1	5.0
Music therapy	2	9.5
Exercise		
Yoga	1	5.0
Naturopathy	5	25.0
Homeopathy	5	23.8
Other, HANDLE therapy, sonic learning, crystals, bush flower essences	10	47.6

^a^Multiple modalities could be nominated

Three main themes were identified in the thematic analysis. First theme was ‘Parents’ experiences of researching CAM treatments’, the second theme was, ‘Navigating CAM information and practices’, which comprises of the subthemes 1) Assessing information on CAM treatments- ‘What counts as ‘evidence’? and 2) Assessing the impact of CAM treatments on the child -‘What counts as effective?, and the final theme was, Creating a central and trustworthy source about CAM.

### Parents’ experiences of researching CAM treatments

For most parents, “doing research” (i.e. researching CAM treatments) was described as complex as it involved searching for a variety of treatments for a combination of ASD symptoms that were affecting their child (e.g. gut issues, nutritional deficiencies and behavioural management). Many parents reported feeling like they were required to take up the position of a ‘lay researcher’ in order to understand potential CAM treatments and the implications of these treatments for their children’s ASD. This was exemplified by Claire who explained:

So basically, I don’t have any science or medical training, but it feels you literally are studying something that way when you start this journey. That’s how it feels. So – yeah, it’s quite - it’s overwhelming.

Most parents stated that they had engaged in research to understand ASD, how it affected their child, and to find appropriate interventions for their child. When parents described how they researched ASD and the treatment options, some parents referred to “the beginning” - the time of their child’s diagnosis. Several parents alluded to being alone in the beginning, overwhelmed or without direction in how to manage their child’s autism as was the case with Olivia, “Gosh, if someone could have handed me an info kit when my son was diagnosed, just on all things that people are doing out there rather than just being – Yeah, it’s a really lonely time”. Olivia’s account suggests that she was given little information regarding treatment options at the time of her son’s diagnosis, resulting in her feeling unsupported and alone. Similarly, Tania talked about the absence of information that was given to her, particularly regarding CAM.

“In those awful early days … there’s a lot of information to take in but I didn’t even really know what complementary medicine was when we started all this so it would be good to understand that from the beginning”.

Furthermore, one mother (Imogen) said that researching CAM treatments was not an easy process, especially in addition to the day-to-day responsibilities that are associated with caring for a child with ASD. Imogen said,

I don’t know if I’m your typical autism mother. There are a few of us out there that are always researching, but most mothers are so tired of just dealing with autism that they don’t even have an inch left to give to researching or reading a book.

These accounts suggest that in light of caring duties, it may be difficult for many parents to fully explore CAM treatment options for their child due to the restrictions on their time and energy, as well as the complexity of researching treatments.

### Navigating CAM information and practices

#### Assessing information on CAM treatments. What counts as ‘evidence’?

Parents talked about accessing a wide variety of information sources to research CAM treatments including books, magazines, DVDs, social media (i.e. Facebook), “research articles”, ASD-related workshops, CAM health practitioners, medical and allied health practitioners, community organisations, online blogs and forums, support groups and other parents. Almost all parents mentioned using the Internet to find information on CAM treatments. For example, Amirtha noted:

“Actually, my husband did a little bit of research on the nutritional, vitamins and stuff. And he found it on the internet and ordered this book which had about all this alternative therapy medicine. And then, we ordered some of the stuff online, and some of the stuff we bought over the counter, some of the mineral supplements, and especially the fish oil and stuff”.

In addition to the variety of sources, parents reported that they were often required to search through vast amounts of information on CAM, which was described as challenging and time consuming. As Cheryl said, “It’s simply because having the time to read everything, you just don’t have it”. Further, much of the CAM information that parents accessed was described by some parents as conflicting. As Tania said, “[there are] a lot of conflicting reports, conflicting anecdotes, conflicting feelings from people about doing it and doing it intensively there’s a lot of negativity about it”. These accounts suggest that researching CAM treatments for their child could be a significant and challenging undertaking for many parents.

Mainstream healthcare practitioners were talked about as a source of CAM information and expertise by most parents. For example, Lydia said that her child’s psychologist was very supportive of CAM, saying, “She does a lot of research, too, and she will mention bits and pieces to me every now and then”. A few participants recounted similar experiences with their general practitioners (GP). In Aimee’s case, her GP encouraged her to alter her son’s diet: “[The GP has] got a child with autism too and she was telling me, ‘Maybe you should try the diet, gluten-free and dairy-free diet’”. However, this was not the experience of all parents. Many parents gave reports of feeling unsupported or uninformed by their GP, specialist or other healthcare professional regarding CAM use with their child. Indeed, some parents were explicitly advised against the use of CAM on the basis that there was no evidence to support its effectiveness for ASD. For example, Amirtha said,

We’re going ahead with the gluten-free/casein-free diet. That’s our choice because we don’t see any harm for the child. My paediatrician didn’t wanna try it. He didn’t want us to do anything. And he even laughed at all this.

The parents’ accounts suggest that while mainstream health practitioners can indeed be a useful source of information for parents, not all health professionals provide information on CAM, or support parents who choose to use CAM with their child. This may limit the amount of evidence-based CAM information that is received by parents from mainstream health practitioners and discourage parents from disclosing CAM use to them.

A small number of parents described the detailed process they used to investigate and examine the legitimacy of, not only the treatment method itself, but the practitioner or supplier of that treatment. For example, Patricia said:

“I’m just looking like right now, looking the wheat grass site. And this guy, which reckons he’s got a qualification, he’s got testimonials on there, there’s contact details on it, that’s really important. If I can’t find out where someone is, then I question that validity. I need to know that they’ve got a real location, they’re a real person. He has got some research on here. ..but [I] don’t jump in straightaway and do [the treatment]. I will follow that for a little while and I will follow the person and read some of their interviews and to see if they’re consistent as well and not just a sort of pop up overnight thing and then they disappear”.

In addition to the process of assessing credibility, another participant described the need to remain “sceptical” when making decisions about CAM treatments. She said:

“So I think there is also a lot of misinformation. There are a lot of people out there who claim to be practitioners of one diet or another and their qualifications may or may not be as good as you think they are.” (Mariana).

These accounts suggest that parents do not necessarily accept information on CAM treatments, practitioners or suppliers at face value but may constantly question the legitimacy and validity of the claims associated this information. Such actions can contribute to the challenging and time consuming nature of seeking reliable information on CAM for parents.

Some parents talked about searching for scientific research as a form of evidence for CAM treatments. However, there was some lack of clarity among parents as to what counted as: scientific evidence”. One parent, Amirtha, referred to this as “solid research”. She said, “Of course, the solid research done by professionals, with case studies and the statistics … That’s what we’re looking for and also scientific research like published scientific research in journals”. Some parents referred to “research papers from Universities” (Olivia) or “stuff that’s being done at a research level at Universities” (Marina) to identify reliable evidence. One participant reporting getting information from “a scientist”, while another participant, Robert, talked about searching for evidence from “clinical trials” or trying to identify “some sort of uniformity in the way they were carrying out the research or testing”. These accounts suggest that while there may be some lack of clarity, parents are searching for evidence that is deemed scientific through the professional or Institutional association of the source and thus deemed to be credible.

Some parents talked about using more than one information source, with scientific evidence combined with testimonials from other parents preferred when considering evidence for treatment options. Testimonials from other parents were often described as “valuable”. This was exemplified by Jacinda, who said, “for me, looking at the scientific part of things is obviously quite important because I’m not just going to go and change my children’s lives for something that doesn’t have any results attached to it or anything like that”. Jacinda also talked about the importance of other parents’ experiences with CAM: “four of five different mothers [were] saying, “Yes, it worked for us. It was great,” all that for me sells it more than a rep or somebody coming and giving me a talk on it”. For some parents, testimonials from other parents were the main source of information and evidence that a CAM treatment could work for their child. Simply knowing that an intervention was successful with another child through word of mouth, was enough to compel parents to try that intervention. For example, Sarah said:

“I knew people who had tried certain things and kind of went, “Oh. That’s it,” and you like you hear that someone else has had success with it. That definitely helps and that - it helps to have a referral to a good person as well.”

Across accounts, testimonials from other parents were trusted and highly valued sources of information either in combination with scientific evidence or as isolated information.

Some parents mentioned using their personal judgements through “gut instinct”, “intuition” and “common sense” when deciding to take-up, continue or discontinue certain CAMs. For example, Patricia said, “I just had to follow my instincts and just go with my gut and if I reasonably think that it makes a lot of sense to me and feels right, then I might follow that through”. In Rob’s case, the feeling that he was being “conned” prompted him to discontinue using a particular naturopath:

I like to think I’m pretty discerning and again how do I prove that to you, I don’t know, you’d have to take my word for it ....there’s a naturopath in [Sydney suburb] who had exorbitant fees ...I believe that he knew what he was talking about but I just felt conned by him so I stopped. But I think that goes for everything. I’ve been given dodgy advice by mainstream medicine as well.

As part of using personal judgement and “common sense” many parents described prioritising the avoidance of any harm to their child in their decision-making regarding CAM treatments. This was often described as a key criterion for CAM use. As Amirtha said, “if this particular alternative medicine or any other therapy or any other medication does not harm the child, we will try it.” In these accounts, the participants took up an active role as ‘gatekeepers’ in their child’s use of CAM, making decisions based on their own personal judgements of intuition and common sense as parents.

When researching CAM treatments, having “proof” or “evidence” of intervention’s efficacy was deemed essential by all the parents. However, accounts of what constituted ‘evidence’ differed among parents. Further, parents often accepted multiple sources of information as evidence.

#### Assessing the impact of CAM treatments on the child - what counts as effective?

Some parents described going through a process of “trial and error” when using CAM with their child to determine whether treatments were effective or not. This involved parents’ employing the use of CAM treatments with their child, monitoring their child’s progress and then adjusting or terminating CAM treatments if they deemed necessary. Cheryl described this process in the following way:

“ … … sometimes it’s just trial and error. Sometimes you just have to sort of take a leap of faith or so and just put the money where - just try it and see whether it does something or not. Sometimes if I’m going into something and I feel like it’s maybe not quite right and sometimes I just push through and do it anyway and I walk out of there and “I knew I shouldn’t have wasted my money on that,” you know what I mean?

As part of trialling CAM treatments, some parents spoke about first trying the treatment on themselves to ensure there would be no harm to their child. As Claire said, “more often than not, I will try the things on me first, before I’ll let them go near my children”. In these accounts, parents took an active role in the administration of CAM treatments trialling, adjusting and ceasing CAM treatments depending on the outcomes they observed.

Many parents described notable and positive changes in their child, which they attributed to CAM interventions. Positive changes in children that were reported by parents included improved behaviour, being calmer, having greater concentration, increased communication, better eating habits, and improved digestion. In most cases, “progress” and positive responses to treatment were described as small improvements. For example, Stella said, “As long as he just gets a bit better all the time and he might sit and draw something or pick up the pen, then that’s progress for me. So just keep doing what we’re doing”.

Most parents detailed the ways in which they documented the effectiveness of CAM treatments to track any impact on their child. For example, Victoria described how she would keep a diary to document the changes in her son’s diet that were working and which were not:

“Making sure that dairy – we sort of tried to eliminate foods when he was very young. … all the certain things that were setting him off. We watched [him] very closely through his behaviour, which would go out with certain food.”

Similarly, Olivia described systematically documenting the positive and negative side effects of each treatment to monitor its effectiveness:

“Well, usually I make a list of what all the negative side effects could be and what all the positive side effects could be and whether I think – I put a question mark next to the ones that I think are doubtful, which is often. And it might take me six months to figure out whether something is working for my son or not”.

The parents’ accounts suggest that they were not complacent in their child’s wellbeing when administering CAM treatments. Rather, parents took an active role in appraising their child’s improvement through documentation and observation to consider whether a treatment was effective and if they should continue the use of that treatment with their child.

CAM treatments were not only assessed by parents in terms of a beneficial impact on their child’s ASD symptoms. For example, some parents described changing their child’s diet by eliminating gluten and diary, which resulted in positive changes for their child. However, making such changes in their child’s diet was deemed too impractical for some parents to maintain, particularly when caring for other family members. As Claire explained:

“So it was that hyperactivity and the behavioural changes that were quite dramatic. So we tried a lot of – just going cold turkey with really everything in the house and really tried the gluten-free and the dairy-free, but he just didn’t eat so we stopped that”.

Although Claire attributed her child’s hyperactivity to the dairy and gluten in her child’s diet, she could not continue with this diet, as it would mean her child would go without food completely. The cost of CAM treatments was also considered an important issue to some parents: “All this stuff does cost money, and money’s always tight when you’ve got other things to consider in the bigger picture with a child with special needs (Olivia)”. Accordingly, parents took into consideration additional factors when assessing the benefits of CAM treatments, such as the child’s reaction to certain interventions and their capacity to maintain the treatment.

In contrast to positive accounts of CAM use, some parents described no improvements in their child’s ASD, while some parents described instances being unsure as to whether CAM treatments were having any impact at all. However, despite being unsure of its effectiveness, some parents talked about continuing to use CAM treatments. For example, Tania described how she kept giving supplements to her child despite not seeing any changes as she was afraid he may be malnourished due to his sensitivities to food. She said, “No, I can’t see a difference between not having them and having them. It’s out of fear that I guess I keep going with vitamin supplements cause I think he’s still really skinny”. Some parents also acknowledged that “every child [is] different”, implying that individual differences between children with ASD may explain why CAM treatments are not always effective. For example, Victoria said:

“As I said I think each child is different and each child requires different things. What my child might be seeking out from a particular therapy program might be truly different to what someone else is seeking for. Because we’re - because the children are also different and have different needs, it’s not like an asthmatic, who just needs a Ventolin spray.”

This meant that some parents reported continuing to seek CAM treatments that were effective for their individual child, despite prior experiences that were unsuccessful.

### Creating a central and trustworthy source about CAM

Overall, most parents reported wanting easier access to quality information on CAM treatments for their child’s ASD. In particular, almost all parents made some reference to the wish for an all-encompassing website, service or repository that contained quality information on CAM treatment options to support their decision-making. For example, Victoria referred to this preferred information base as a “one stop shop”:

“You would have a universal website, like a one stop shop, where you can get everything. So instead of trying to go on ten different websites, I [think] probably have one. I’d have list of every business in [name of place] where a parent could just click on it and have a blurb about it. And then it would have link going to that service’s website. I think the website should have an introduction on what is alternative therapy and how can you use it”.

Similar to the descriptions from other parents, the key features in Victoria’s suggestion are that CAM information is centralised, easy to access and user friendly. In this vein, Mariana suggested that parents could have access to a “cheat sheet” on CAM information that presented detailed information regarding the method for using a CAM treatment and the efficacy of that treatment. In addition, many parents emphasised the importance of trustworthy information. As Tania said, “I guess if there was a central place that you could go, a trusted, reputable, independent place, source that you could get information from that would be a help. These accounts suggest that convenience, efficiency, and trustworthiness of information on CAM are important factors in addressing many of the challenges that parents experience when researching CAM treatments.

## Discussion

This study explored the views and perspectives on CAM decision-making among parents of children with ASD, including how they research treatments and what is accepted as ‘evidence’ and ‘effectiveness’ of CAM treatments. Parent’s decisions to take up and maintain CAM methods related to time, potential of harm for the child, cost and practicality of administering the treatment. All of the parents described taking up an active rather than a passive role in relation to their children’s health. In particular, all of the parents reported being engaged in active researching CAM treatments for ASD. However, the sources of information used and the way in which parents assessed the credibility of information and efficacy of CAM treatments varied greatly.

The majority of parents engaged in in-depth research of treatment, trying to understand what treatments are out there and what may be beneficial to their child. It seems that they were navigating their way through a vast amount of complex information, which can often be conflicting, particularly in relation to the effectiveness of the treatment or the credibility of the source. As such, parents reported using a combination of filters to ascertain whether a certain CAM was worth trialling with their child, such as scientific evidence, expert opinion, testimonials from parents, “gut instinct” and trial and error. Similar to our results the ‘trial and error’ method to choosing and evaluating general ASD interventions was reported by a qualitative study of parents of children with ASD. In this study parents were not able to accurately explain the term “evidence-based” and did not usually consider whether an intervention had research to support its effectiveness [22].

In our study the types of information used by parents in their decision making was wide ranging. While some parents placed value on scientific evidence others balanced this information with testimonials from other parents who had children with ASD. Studies have also shown that parents rely upon “word of mouth” (i.e., other parents’ recommendations) [10, 37] A systematic review exploring the factors related to parents’ treatment decisions for their children with ASD described that recommendations from others such as other parents, relatives and friend are all rated important by parents [38]. However, this poses a dilemma for parents; if there is some evidence that a CAM is effective for certain children with autism, parents may be compelled to try it, provided it does not harm their child. Therefore, even if treatments have only been successful in a small percentage of cases, some parents may still consider trialling that treatment in the hope that it will be successful for their child. Hebert [39] described how parents making treatment decisions for children with ASD take responsibility of making a decision on behalf of their child very seriously. They are fearful of making the wrong decision and see it as their parental role to be certain that the best decision is made. These decisions may be more challenging when considering CAM, because evidence is less adequate than desired or may be lacking [40]. Parents may make decisions in conditions of uncertainty [41].

Our study also found that some parents disclose the use of CAM to health care providers (HCPs) and others do not. Research as described the reasons why parents do not disclose CAM use include: the perception that HCPs are not knowledgeable about CAM treatments, they don’t ask about them or because they are concerned their decision to use CAM treatments in their children will cause conflict with their HCPs [42–44]. O’Keefee and Coat [45] also reported bad experiences with doctors when parents discussed CAM use. As noted by Albert [46], HCMs should keep an open mind and be nonjudgmental when patients and parents wish to discuss CAM, and they should be knowledgeable about CAM, the values and beliefs of various cultures, and the specific health related practices and values of their own patients. The American Academy of Pediatrics (AAP) Committee on Children with Disabilities guidelines for physicians discussing the use of CAM treatments with their patients and families [47].

A systematic review by Lorenc et al. [48] aimed to identify a conceptual framework which can successfully model for the parental decision-making process of choosing to use CAM for children. The review found that the Andersen’s sociobehavioural model (SBM) has been the most widely use and some authors identify it as a suitable one to describe the decision-making process resulting in adult CAM use. However, the review also found that the appropriateness of its application to child CAM use has not fully been studied and needs further clarification. Sirois et al. [49] note that the SBM does not account for the more complex nonlinear processes that may determine why and how decisions between subgroups of CAM users differ. They propose a consumer decision-making model as it takes into account the diversity of decision factors influencing the decision to use CAM. However, this model does not include factors relating to interactive and emotional aspects of care which may be important for both CAM use and child healthcare and is not specifically related to health [48]. Further research is needed to understand the decision making process in child CAM use. Especially considering use of CAM with children with ASD may be complex as children do not usually decide about treatment for themselves.

The present analysis demonstrates the significant difficulties that parents experience when seeking information on treatment options for ASD. These findings suggest that parents would welcome any support to make the process of caring for their child with ASD less stressful, reduce the risk of harm to their child’s mental and physical wellbeing, while also being less consuming of their time, money and energy. This highlights the need for the development of an accessible up to date evidence based resource on CAM that is co-designed with key parent and carer and autism stakeholder groups. As described by Gilmour et al. [41], have the greatest interest in their children’s well-being, know their children best, and carry the greatest burden if things do not go well. Decision-making may be more challenging when considering CAM, because evidence may be lacking or less adequate than desired [40].. Frameworks to guide both parents and health professionals who care for children with ASD that support treatment decisions are needed [41]. High quality clinical studies on many CAM to manage symptoms of autism are lacking and these studies are a priority to guide and inform evidence-based resources.

As reported previously [20], parents’ self-rated effectiveness of CAM for improving their child’s autism symptoms was higher for sensory integration therapy, melatonin, and off-label use of prescription antifungal,. This is despite the limited evidence of effectiveness of these therapies [13, 50]. These findings highlight the importance of providing quality evidence to determine CAM level of safety and efficacy. Having access to reliable information is a particular challenge for parents who are caring for a child with ASD, which often renders parents time-poor and exhausted. Therefore, measures to make the research process easier for parents, such as increased accessibility and increased credibility would provide a positive and useful element in not only the parent’s lives, but their children’s lives as well.

In Canada the Complementary Medicine Education and Outcomes (CAMEO) Research Program has developed tools to support people living with cancer in making evidence-informed decisions about CAM use and are exploring ways to improve health professionals’ knowledge and decision-support skills related to CAM [51]. In Australia an adapted version of the instruments is currently being trialled with people over 65 years who may or may not be CAM users [52] . Even though the focus of these resources is not on children with ASD these tools could potentially be adapted to provide education to parents/cares of children with ASD about CAM and enhancing their skills and knowledge to evaluate CAM evidence, risk to benefit and ultimately advise on safety and contraindications. This is supported by research findings from a qualitative study with parents of children with ASD by Grant et al. [22]. The study described the complexities of the decision-making processes and information preferences of parents of children with ASD but also the need for these parents to be supported to make informed decisions especially around research evidence. Future research could explore the effectiveness of these and other tools that support CAM decision making and help parents understand research evidence.

### Strengths and limitations

The number of parents interviewed for this study was small, however thematic saturation of data was achieved. As such, as an exploratory qualitative study it is possible that the views of our study participants were not representative of parents and carers of children with ASD more generally. In addition, the majority of the participants were predominantly Caucasian, and tertiary educated, and their perspectives may not necessarily reflect those of lower socio-economic backgrounds or diverse cultural groups. Since conducting this study the National Disability Insurance Scheme (NDIS) has been implemented and provides support to eligible people with intellectual, physical, sensory, cognitive and psychosocial disability. It is possible that some of the support parents are now able to access my address some of the findings highlighted in this study. Our sample also reflected a group of parents who were heavily reliant on the use of internet and, therefore, our findings may not reflect parent’s views and experiences who draw more heavily on other information sources, including those who might rely predominantly on information from mainstream health professionals. Additionally, the parents in our study were managing a broad range of complex ASD symptoms and levels of severity. Future research could examine the way that parents navigate CAM-related information on ASD in more depth by examining the influence of ASD severity on parents’ experiences and decision-making regarding CAM. Research can also explore how the decision-making process changes based on the time since ASD diagnosis. While this study asked about the age the child was diagnosed it did not account for the time since diagnosis. However, our study complements the existing CAM related knowledge base by drawing on parent’s accounts of their experiences and decision-making around CAM-related information. Further research examining the information needs of parents of children with autism post the introduction of the NDIS is recommended.

## Conclusion

This study found that parents of children with ASD are actively seek out information on complementary health approaches to assist with their child’s ASD symptoms. Navigating information to identify credible information was complex and time consuming. Information was fragmented and varied in quality. This contributed to participants relying on a variety of information sources and gut instinct to establish a case that CM treatments worked and benefitted to their children. An understanding of the term evidence was lacking. These findings highlighted opportunities to address the health literacy skills of parents of children with autism, the need for high quality clinical research on the effectiveness of treatments and the translation of this research to inform the development of reliable and up to date web resources.

## Data Availability

The datasets used and/or analysed during the current study are available from the corresponding author on reasonable request.

## Abbreviations

ASD: Autism spectrum disorder
CAM: Complementary and alternative medicine
CAMEO: Complementary Medicine Education and Outcomes
GFCF: Gluten-Free, Casein-Free Diet
GP: General practitioners
NDIS: National Disability Insurance Scheme
NHMRC: National Health and Medical Research Council
